# Comparative analysis of the metabolite compositions and antioxidant capacity of waxy and non-waxy black rice (*Oryza sativa* L.) bran via widely targeted metabolomics

**DOI:** 10.1016/j.fochx.2025.103387

**Published:** 2025-12-08

**Authors:** Shiquan Bian, Shaojie Song, Mingding Qu, Xiaojing Dang, Deyong Mei, Cuixiang Lin, Wanlin Wang, Jintao Shang, Dahu Ni, Dewen Zhang

**Affiliations:** a Rice Research Institute, Anhui Academy of Agricultural Sciences, Hefei 230031, China; b Key Laboratory of Rice Germplasm Innovation and Molecular Improvement of Anhui Province, Hefei 230031, China; c Agricultural Technology Extension Center of Linping District, Hangzhou 311199, China

**Keywords:** Black rice, Metabolite, Antioxidant capacity, Widely-targeted metabolome analysis, Polyphenol compounds

## Abstract

Black rice (*Oryza sativa* L.), valued for its nutritional benefits, was investigated to elucidate metabolite profiles and antioxidant capacity differences between waxy and non-waxy varieties. Eight black rice varieties sourced from diverse Chinese provinces were categorized based on amylose content. Non-waxy varieties demonstrated significantly superior antioxidant capacity, evidenced by elevated levels of DPPH and ABTS radical-scavenging activities, FRAP values, total polyphenol content, and α-glucosidase inhibitory activity. Utilizing UPLC-MS/MS, a total of 1130 secondary metabolites were identified, with flavonoids and phenolic acids constituting prominent classes. Multivariate analysis revealed distinct metabolite profile differences between the waxy and non-waxy groups, particularly in associated metabolic and genetic pathways. The enhanced antioxidant capacity and α-glucosidase inhibitory activity were strongly associated with differential metabolites, notably flavonoids. These findings underscore the potential of non-waxy black rice bran components, particularly flavonoid-rich fractions, for development in functional food applications. .

## Highlights

• Non-waxy black rice bran exhibits significantly higher antioxidant capacity (DPPH, ABTS, FRAP), total polyphenols, and α-glucosidase inhibitory activity compared to waxy bran.
• Widely targeted UPLC-MS/MS metabolomics identified 1130 metabolites in black rice bran, with flavonoids and phenolic acids as major classes.
• Multivariate and association analyses revealed distinct metabolite profiles between waxy/non-waxy groups and linked the enhanced functional properties in non-waxy bran specifically to differential flavonoids.
• Identification of key differential metabolites provides a foundation for targeted development of black rice bran components and breeding improved varieties for functional food applications.

## 1 Introduction

Rice (*Oryza sativa* L.) is a major edible grain cereal and belongs to the grass family. As a basic staple food, it supplies nutrition to nearly half of the population of the world (Jing et al., 2023). In addition to being a staple source of energy, rice possesses a great pool of bioactive phytochemicals, and the endogenous metabolites are abundantly concentrated in milling fractions, such as the bran and the germ (Tan et al., 2023). As rice bran, equivalent to 8–12 % of rice paddies weight as a layer between the starch-endosperm and the husk, is no longer a low value by-product but rather a rich functional ingredient (Pang et al., 2018). It is the major source of different types of bioactive compounds such as polyphenols, tocopherols, tocotrienols and γ -oryzanol, all of which together account for many health promoting attributes such as antioxidative, anti-carcinogenic, and hypolipidemic activities (Kodape et al., 2025).

The dark pericarp colour in pigmented black rice arises from a high level of the antioxidants anthocyanins, polyphenols and other flavonoids and has garnered great scientific and commercial attention due to its high oxidative potential (Chen et al., 2024). This grain has a long history of food and use in therapeutic applications throughout numerous Asian nations including China, Japan, South Korea, and Thailand, where it is acknowledged as a nutritious food (Yang et al., 2025). The potential health benefits of black rice attributed mainly to its bran-derived bioactive compounds (such as anthocyanins and γ- oryzanol), include the effects of decreasing insulin resistance, reducing cholesterol absorption, limiting mammary carcinogenesis, and delaying senescence accelerated by D-galactose (Cheng et al., 2024; Laorodphun et al., 2024; Nafisah et al., 2023).

Stratified milling has previously been shown to produce a clear radial progression of different bioactive compounds in black rice bran. It was reported that the pericarp layer with the highest concentration of flavonoids, phenolic acid, γ-oryzanol, vitamin E isoforms, and anthocyanins, dominated by cyanidin-3-O-glucoside (the most abundant of black rice anthocyanins, accounting for 90 % of total black rice anthocyanins) (Kraithong et al., 2025; Zhang et al., 2023). An in vitro and in silico screening both established cyanidin-3-glucoside and 6’-O-feruloylsucrose as two new high potent inhibitors of α-glucosidase (Bhuyan et al., 2022), which put forward the ability of black rice bran exerting its health benefit owing to its abundance of flavonoids and phenolics compound and the resulting antioxidative effects.

The major shortcoming of the existing body of black rice studies is that they commonly view black rice as a homogenous group (Borah et al., 2025; Zhang et al., 2023), despite the considerable diversity observed between different cultivars, and the respective variations in starch composition and functional characteristics of black rice cultivars that play a major role in determining its nutritive and functional quality (Oikawa et al., 2015). The separation of black rice into waxy and non-waxy varieties can be mainly traced back to its starch content in terms of amylose content. Waxy black rice is characterized by an amylose content typically below 2 %, resulting in a soft, cohesive texture upon cooking due to the predominance of amylopectin (Adegoke et al., 2021; Tang et al., 2016). Importantly, this different starch architecture could have implications beyond flavour and might alter biosynthesis of bioactive metabolites, as evidenced previously that genetic controls of starch metabolism might control wider sets of metabolism (Baysal et al., 2020; Galland et al., 2017). Nevertheless, the direct regulatory relationship between the starch biosynthesis pathway and the biosynthesis of health-promoting chemical substances in black rice remains largely unexplored. It is important to dissect the relationship between this phenotype and the metabolites for better understanding how this genetic diversity of black rice may determine the functional nutritional value of black rice.

Thus, to this research has attempted to elucidate the secondary metabolic pathways and the associated bioactivities in black rice bran with a focus on how the waxy trait affects them, and we postulated that the waxy trait modified the profiles of bioactive metabolites particularly the flavonoids and phenolic acid so as to also lead to differential antioxidant and α-glucosidase inhibitory capacities. In order to assess this hypothesis, a commonly-targeted UPLC-MS/MS metabolomics approach, including in vitro assays were employed to characterize metabolite composition and biofunctional properties systematically. Furthermore, some important different metabolic pathways were involved further for relating starch genetics to the secondary phytochemistry. The outcomes should be helpful to guide targeted breeding of black rice cultivars that have higher nutritional and health-related values.

## 2 Materials and methods

### 2.1 Plant materials

**Fig. 1:**
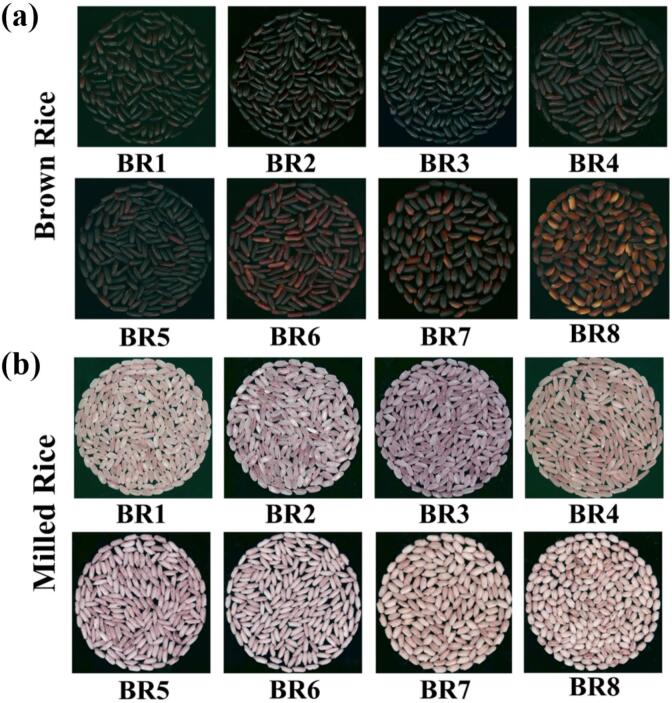
Eight black rice varieties used in this study. (a) brown rice; (b) milled rice. (For interpretation of the references to colour in this figure legend, the reader is referred to the web version of this article.)

A total of eight locally representative black rice varieties from different provinces in China were selected as the experimental materials in this study, namely, BR1 (Yimengheimi), BR2 (Jiagouheimi), BR3 (Heizhenzhu), BR4 (Mojiangzimi), BR5 (Yangxianheigu), BR6 (Bamaheimi), BR7 (Zixiangnuo) and BR8 (Ziquezimi). These cultivars were selected according to their agronomic dominance and commercial representativeness in their regions of origin. As locally dominant cultivars which are well-known by farmers and the food industrial companies ensure the applicability of the results obtained from the germplasm used in the study to crop breeding and functional food development. Sample information is listed in Fig. 1 and Table S1.

All experimental materials were cultivated synchronously during the 2024 growing season under standardized agroecological conditions in the experimental field of Linping Agricultural Technology Extension Center (Hangzhou, China), Post-harvest, paddy grains were subjected to controlled drying at 40 °C in a forced-air oven until achieving a moisture content of 10–11 % (*w*/w), followed by cryopreservation at 4 °C in opaque containers. For metabolomic analysis, samples were dehulled using a THU35C laboratory-scale huller (Satake Suzhou Co., Ltd.) to obtain brown rice, which was subsequently processed via the LTJM-6688 precision milling system (Taizhou Grain Instrument Co., Ltd.) using a 30-s milling protocol optimized to yield approximately 10 % (w/w) fresh rice bran with minimal structural degradation. The experimental design incorporated six independent biological replicates per rice genotype, with all processing parameters rigorously documented to ensure methodological reproducibility.

### 2.2 Determination of amylose content (AC) in rice

Milled black rice was pulverized using an industrial grinder, followed by sieving through a 100-mesh screen. This sample preparation protocol adhered to a previously established methodology (Gaenssle et al., 2021), amylose content in black rice grains was determined with a amylose/amylopectin assay kit (Megazyme, Ireland).

### 2.3 Sample preparation and extraction

The black rice bran samples underwent grinding for 1.5 min (Tissuelyser-LT, Jingxin) followed by full lyophilization using a vacuum freeze dryer (CSIENTZ-10ND, Simon). Freeze-dried powder (10 g) was resuspended in 100 mL of 70 % methanol. Sequential processing involved:(1) Cryo-homogenization at −10 °C (50 Hz, 6 min) using a tissue grinder (Wonbio, CA); (2) Subzero ultrasonic extraction (5 °C, 40 kHz, 30 min). Post-homogenization, samples underwent cryogenic incubation (−20 °C, 30 min) to facilitate precipitation. The suspension was centrifuged (13,000 ×*g*, 15 min, 4 °C), with the clarified supernatant preserved at 4 °C pending biological and UPLC-MS/MS analyses. For analytical quality assurance, a pooled quality control (QC) sample was generated by combining 20 μL aliquots from each extract. This QC benchmark was interspersed after every six consecutive experimental injections to validate analytical stability and methodological precision throughout the sequence.

### 2.4 Determinations of antioxidant capacity in black rice bran samples

#### 2.4.1 Determination of free radical scavenging activity

2,2-diphenyl-1-picrylhydrazyl (DPPH) radical scavenging activity was measured following Shang et al.'s method (Shang et al., 2019). For DPPH radical scavenging assessment, 20 μL extract was reacted with 180 μL DPPH reagent (32 μg/mL). Following 30-min incubation (25 °C), absorbance was measured at λ = 517 nm. A quercetin-based calibration curve (y = 3.697× - 4.315; 0–160 μg/mL, *n* = 6, R^2^ = 0.9996) enabled quantification of antioxidant capacity as quercetin equivalents per gram dry weight (mg QE/g DW). ABTS^+^ analysis followed Shang et al.'s methodology (Shang et al., 2019). The sample extract was reacted with 180 μL ABTS reagent. Absorbance measurements (734 nm) followed 20-min incubation. Quercetin served as the reference standard, generating a calibration curve (y = 3.807× - 3.6447; R^2^ = 0.9992) across 0–160 μg/mL (n = 6). Antioxidant capacities were normalized to milligrams quercetin equivalents per gram dry weight (mg QE/g DW), with six biological replications per sample.

#### 2.4.2 Determination of ferric-reducing antioxidant power assay

Ferric-reducing antioxidant power (FRAP) was assessed according to established methods (Romero-Márquez et al., 2024). Briefly, sample extracts (20 μL) reacted with FRAP working solution (265 μL) and ultrapure water (15 μL). Post-incubation (37 °C, 30 min), absorbance was recorded at λ = 595 nm. A six-point standard curve established with FeSO₄·7H₂O (y = 0.2904× + 0.1269, R^2^ = 0.9993) enabled quantification of total antioxidant capacity as micromolar FeSO₄ equivalents per gram dry weight (μmol/g DW).

### 2.5 Determinations of the total polyphenol contents in black rice bran samples

Referring to the method proposed by Shang et al. (Shang et al., 2019), Total polyphenols in black rice bran were quantified via Folin-Ciocalteu assay. A six-point calibration curve generated with gallic acid standards yielded the regression equation y = 2.0601× + 0.675 (R^2^ = 0.9995). Results were expressed as gallic acid equivalents (GAE) per gram dry weight (mg GAE/g DW).

### 2.6 Determinations of α-glucosidase inhibitory activity in black rice bran samples

α-Glucosidase inhibitory activity was assessed per Lv et al. (Lv et al., 2021). Black rice bran extracts (0.4 mg/mL in 100 mmol/L PBS, pH 6.8) were analyzed in 96-well plates containing: 40 μL PBS, 20 μL extract, 20 μL α-glucosidase (0.5 U/mL). After 15-min incubation (37 °C), 20 μL PNPG (5 mmol/L) was added, followed by additional 10-min reaction. Termination used 100 μL Na₂CO₃ (0.2 mol/L) with absorbance measurement at λ = 405 nm.

$$\text{Inhibition}\;\left(\%\right)=\left[1–\left(A\;\text{sample}-A\;\text{sample blank}\right)/\left(A\;\text{control}-A\;\text{blank}\right)\right]\times 100\%$$

### 2.7 Ultra-performance liquid chromatography-mass spectrometry (UPLC-MS) detection analysis condition

Metabolite separation and detection employed an UHPLC-Q Exactive system (Thermo Scientific, USA) equipped with an ACQUITY UPLC BEH C18 column (100 × 2.1 mm, 1.7 μm; Waters, USA) at 40 °C. The mobile phase comprised: (A) 2 % acetonitrile/0.1 % formic acid aqueous solution and (B) acetonitrile/0.1 % formic acid, with 3 μL injection volume. Separation used the gradient program detailed in Table S2.

Samples were ionized using a heated electrospray ionization (HESI) source in dual polarity mode, enabling simultaneous positive/negative ion (ESI±) data acquisition. Detailed instrumental parameters are provided in Supplementary Table S3. For quality control (QC), pooled matrix specimens were prepared by combining equal aliquots of all experimental extracts and processed identically to study samples. QC injections occurred systematically every 5–15 samples to monitor instrument stability and chromatographic reproducibility.

### 2.8 Identification of compounds and peak area extraction

Raw metabolomic datasets were processed using Progenesis QI v3.0 (Waters Corporation, Milford, MA, USA) through a standardized pipeline encompassing baseline subtraction, peak picking, integration, retention time normalization, and chromatographic alignment. This computational workflow generated a quantitative data matrix encoding retention time (RT), mass-to-charge ratio (*m*/*z*), and peak intensity values for all detected features. For metabolite annotation, the software performed automated spectral library searching against the MJDBPM plant-specific metabolite database (Majorbio Bio-Pharm Technology Co., Ltd., Shanghai, China) using both MS^1^ and MS^2^ fragmentation data. Precursor ion mass accuracy was constrained to ≤10 ppm, with additional validation achieved through spectral matching confidence scoring of MS/MS fragmentation patterns against reference library entries.

## 3 qRT-PCR analysis

The total RNA was extracted from the rice bran samples through the method of VAMNE Magnetic Universal Total RNA Kit (Vazyme, Nanjing, China) according to the instructions. The first strand cDNA was synthesized from the extracted RNA by the method of HiScript IV All-in-One Ultra RT SuperMix (Vazyme, Nanjing, China). The expression of genes was analyzed by qRT-PCR using the Taq Pro Universal SYBR qPCR Master Mix (Vazyme, Nanjing, China) on an ABI QuantStudio 7 Pro system (Thermo Fisher Scientific, Waltham, MA, USA). Relative expression level was used by 2^-ΔΔ*Ct*^ algorithm according to the previous research (Bian et al., 2022). We created primers with the help of NCBI online tools (http://www.ncbi.nlm.nih.gov/). qRT-PCR primers information is shown in Table S4.

### 3.1 Data processing

Statistical analyses employed MetaboAnalyst 5.0 (https://www.metaboanalyst.ca/), including principal component analysis (PCA) and partial least squares-discriminant analysis (PLS-DA), with pairwise comparisons assessed by Student's *t*-tests. Significantly altered metabolites underwent pathway enrichment analysis using hypergeometric tests against the Plant Metabolic Network (PMN, http://www.plantcyc.org/) and Kyoto Encyclopedia of Genes and Genomes (KEGG) databases. Significantly enriched pathways in which the metabolites in a module were involved were compared to the background and defined by both a hypergeometric test and a threshold of *p*-value <0.05.

## 4 Results and discussion

### 4.1 Phenotypic characteristics of black rice grains

Eight representative local black rice varieties (BR1-BR8) from eight provinces in China were chosen for further research in this study. The harvested black rice was husked to get the brown rice, and then were milled to generate black milled rice finally. The appearance of BR1-BR4 milled rice grains show transparency with less chalkiness, while BR5-BR8 rice grains exhibit completely white and opaque (Fig. 1). Furthermore, the amylose content (AC) of these black rice varieties was examined in this study. As shown in the Fig. S1, the amylose content of BR1-BR4 was 17.25 %, 15.27 %, 14.32 % and 8.46 %, respectively, while amylose content of BR5-BR8 was 1.74 %, 0.62 %, 1.18 % and 1.27 %, respectively. Therefore, we divided BR1-BR8 into two groups, namely non-waxy black rice: BR1, BR2, BR3 and BR4 and waxy black rice: BR5, BR6, BR7 and BR8 (Adegoke et al., 2021).

### 4.2 Antioxidant activity and total polyphenol content of black rice bran samples

**Fig. 2:**
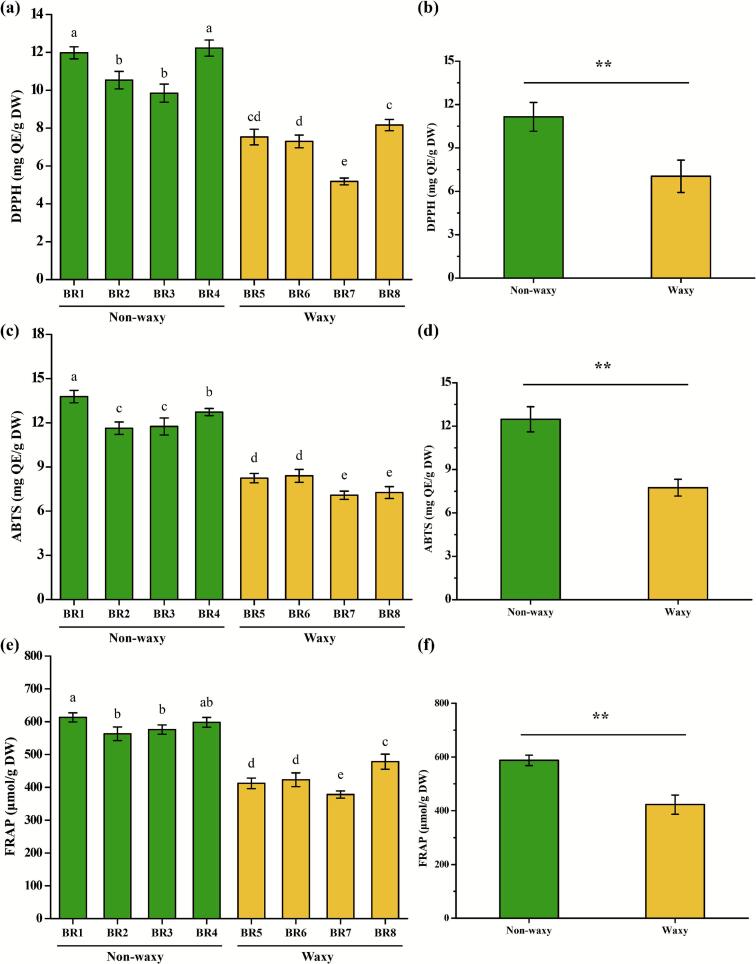
The antioxidant activities of black rice bran samples. (a) the DPPH radical-scavenging activity; (b) the comparison of DPPH radical-scavenging activity between waxy and non-waxy black rice bran; (c) the ABTS radical-scavenging activity; (d) the comparison of ABTS radical-scavenging activity between waxy and non-waxy black rice bran; (e) the FRAP assay; (f) the comparison of FRAP assay between waxy and non-waxy black rice bran. Data sharing the different letters were significantly different (*p* < 0.05), and the ** indicates a significant difference between waxy and non-waxy black rice bran (*p* < 0.01).

Black rice bran extract has been proven to be rich in free phenols, which are closely related to antioxidant activity (Lv et al., 2021). The antioxidant activities of the extract from different black rice bran samples are shown in Fig. 2. Generally, DPPH is considered as a representative reagent for detecting the free radical scavenging activity of plant bioactive compounds (Shang et al., 2019). In this study, BR1 and BR4 exhibited the best scavenging activity, while BR7 showed the worst effect (Fig. 2 a). Compared to waxy black rice bran samples, non-waxy groups showed a 13.13 % increase in the DPPH radical-scavenging capacity (Fig. 2 b). For ABTS radical-scavenging activity of the extract, the activity levels were the following in increasing order: BR7, BR8, BR5, BR6, BR2, BR3, BR4, and BR1 (Fig. 2 c). Specifically, the ABTS radical-scavenging activity of the non-waxy groups was significantly higher than that of waxy groups, increasing from 7.74 ± 0.58 mg QE/g DW to 12.47 ± 0.87 mg QE/g DW (Fig. 2 d). Moreover, the FRAP assay of non-waxy black rice bran significantly enhanced compared with waxy groups (Fig. 2 e, f). Based on the above results, it can be preliminarily concluded that the extract from non-waxy black rice bran exhibited excellent antioxidant capacity, thus possibly acting as primary antioxidants (Lv et al., 2021; Shang et al., 2019).

**Fig. 3:**
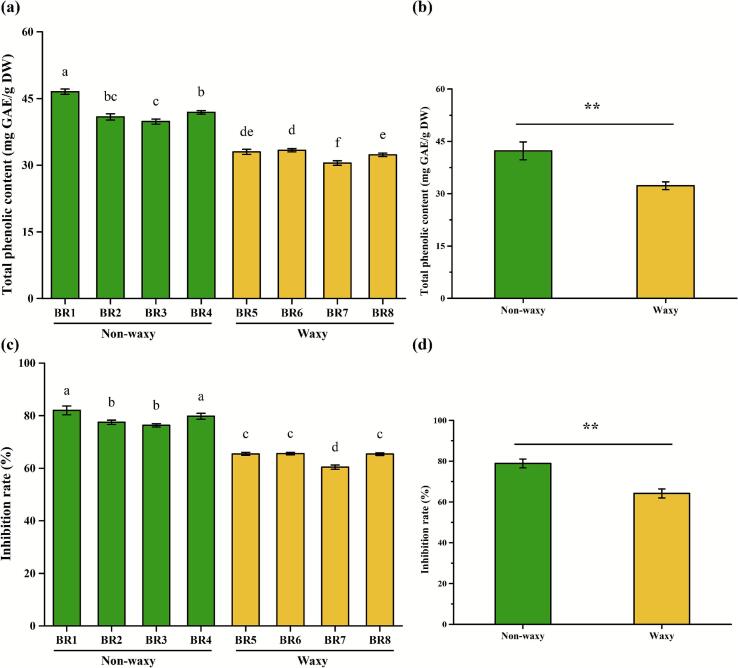
The total polyphenol contents and α-glucosidase inhibitory activity of black rice bran samples. (a) the total polyphenol contents; (b) the comparison of total polyphenol contents between waxy and non-waxy black rice bran; (c) the α-glucosidase inhibitory activity; (d) the comparison of α-glucosidase inhibitory activity between waxy and non-waxy black rice bran; Data sharing the different letters were significantly different (*p* < 0.05), and the ** indicates a significant difference between waxy and non-waxy black rice bran (*p* < 0.01).

Polyphenols, key bioactive constituents of black rice (Yu et al., 2024), were quantified across eight bran varieties (Fig. 3 a). Compared with other black rice bran samples, BR1 possessed higher contents of polyphenols (46.54 ± 0.60 mg GAE/g DW). Non-waxy black rice bran exhibited significantly higher total phenolic content (+30.75 %; *p* < 0.01) compared to waxy varieties (Fig. 3 b). Key phenolic compounds including cyanidin-3-O-glucoside chloride, ferulic acid, protocatechuic acid and vanillic acid demonstrate α-glucosidase inhibitory effects (Akkarachiyasit et al., 2010). The inhibitory rates of BR1, BR2, BR3 and BR4 on α-glucosidase were significantly higher than that of BR5, BR6, BR7 and BR8 (Fig. 3 c). Mai et al. found a positive correlation between the α-glucosidase inhibitory activity and polyphenol content of water and methanol extracts of 28 edible plants (Mai et al., 2007). Consequently, non-waxy bran demonstrated significantly stronger inhibitory activity versus waxy counterparts (p < 0.01, Fig. 3 d), attributable to its elevated phenolic content (Fig. 3 b).

### 4.3 Identification of the metabolites in black rice bran samples

**Fig. 4:**
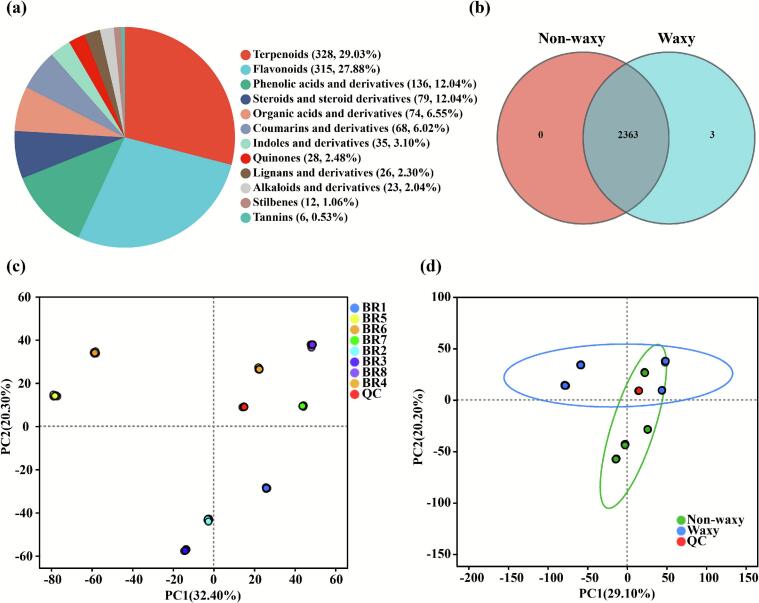
The classification, Venn diagram, and PCA of metabolic profiles in black rice bran samples. (a) the classification of the 1130 metabolites of brown rice samples based on plant secondary metabolites; (b) Venn diagram of shared and unique metabolites in non-waxy and waxy groups; (c) PCA map of metabolic profiles in eight black rice bran samples; (d) PCA map of metabolic profiles in non-waxy and waxy groups. (For interpretation of the references to colour in this figure legend, the reader is referred to the web version of this article.)

The comprehensive metabolic profiling of eight black rice bran samples was conducted by a widely targeted metabolite analysis using UPLC-MS/MS (Fig. S2 and S3). Based on the classification of plant secondary metabolites, a total of 1130 secondary metabolites were identified in this study, including tannins (6, 0.53 %), stilbenes (12, 1.06 %), alkaloids and derivatives (23, 2.04 %), lignans and derivatives (26, 2.30 %), quinones (28, 2.48 %), indoles and derivatives (35, 3.10 %), coumarins and derivatives (68, 6.02 %), organic acids and derivatives (74, 6.55 %), steroids and steroid derivatives (79, 12.04 %), phenolic acids and derivatives (136, 12.04 %), flavonoids (315, 27.88 %), and terpenoids (328, 29.03 %) (Fig. 4 a). Moreover, a total of 2363 metabolites were shared by both waxy and non-waxy groups, with only three compounds unique to waxy black rice bran (Fig. 4 b). Flavonoids and phenolic acids, as important plant active substances, were also detected in all samples, indicating that all eight types of black rice bran have good biological activities (Shao et al., 2018; Zhu et al., 2022). In a word, these analyses revealed consistent metabolite compositions among all groups.

### 4.4 Multivariate statistical analysis of the metabolites in black rice bran samples

Multivariate statistical modeling was applied to eight black rice bran samples categorized into waxy and non-waxy genotype groups to identify metabolomic signatures distinguishing these phenotypically distinct subsets. Specifically, principal component analysis (PCA) was employed to visualize metabolic variations across the sample cohort, enabling assessment of global metabolomic divergence among the eight black rice bran samples while accounting for genotype-specific effects (Fig. 4 c). The highly concentrated character indicated the better repeatability for each black rice bran group. The two principal components (PC1 and PC2) in the PCA score plot explained 31.40 % and 20.30 % of the variance, respectively, indicating remarkable separation between the eight black rice accessions and high cohesion within groups. In addition, the two principal components explained 49.30 % of the total variance (Fig. 4 d). Metabolomic analysis revealed significant compositional divergence between waxy and non-waxy groups. This dichotomy segregated the eight varieties into two discrete clusters, each exhibiting unique biochemical signatures.

**Fig. 5:**
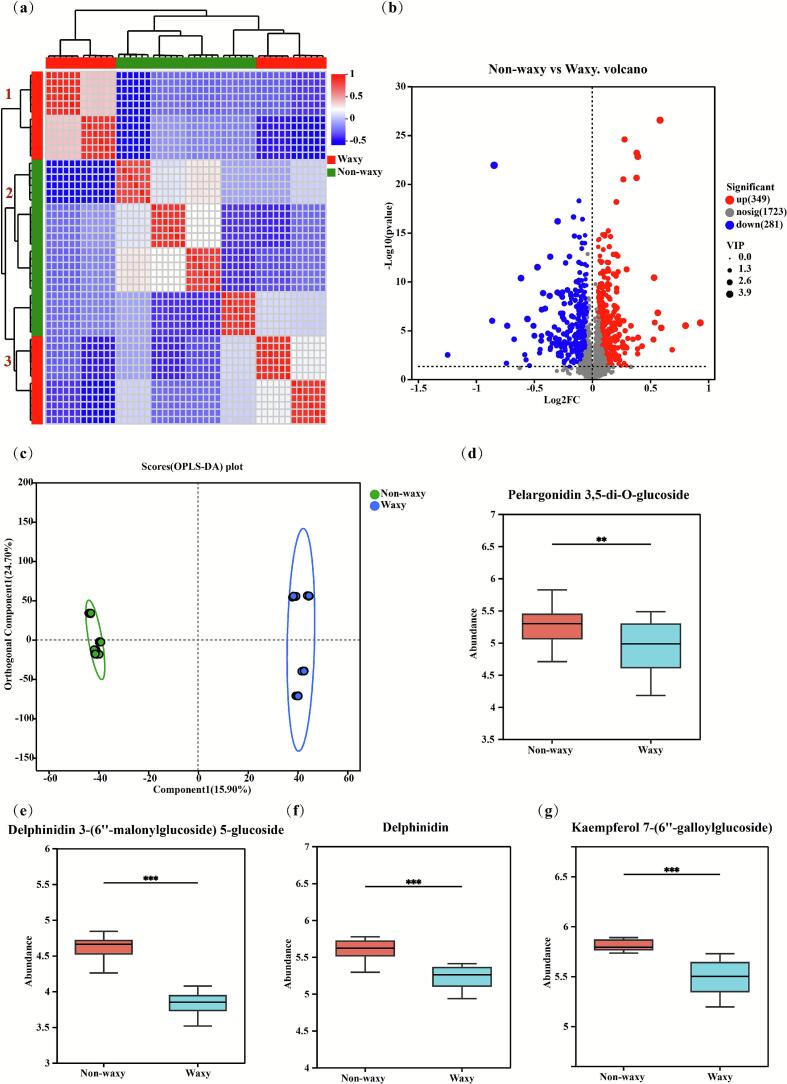
The hierarchical cluster analysis (a), volcano plots (b), score plots generated from OPLS-DA (c), and comparison of metabolic profiles in non-waxy and waxy groups. (d-g) Comparisons of pelargonidin 3,5-di-O-glucoside, delphinidin 3-(6″-malonylglucoside) 5-glucoside, delphinidin, and kaempferol 7-(6″-galloylglucoside) in non-waxy and waxy groups.

Hierarchical clustering identified three statistically significant metabolite clusters exhibiting differential abundance patterns (Fig. 5 a). Metabolites within clusters 1 and 2 exhibited preferential accumulation in waxy and non-waxy groups respectively, confirming divergent compositional landscapes between these cohorts. In addition, the cluster 3 showed high accumulation in both waxy and non-waxy groups. The differential metabolites were selected between waxy and non-waxy groups based on VIP-pred-OPLS-DA (≥ 1) and *P*-value (≤ 0.05). The volcano plots showed that there were 630 significantly different metabolites between non-waxy vs waxy (349 up-regulated and 281 down-regulated) (Fig. 5 b). Employing orthogonal rotation to filter non-group-related variance, the OPLS-DA model (Kang et al., 2022) significantly enhances intergroup discrimination and model robustness. Corresponding score plots revealed clear metabolic segregation between waxy and non-waxy cohorts, indicative of fundamental biochemical divergence in black rice bran (Fig. 5 c).

Extensive evidence confirms that anthocyanin-flavonoid fractions in black rice mediate key pharmacological properties, including antioxidative, hypoglycemic, and anti-inflammatory bioactivities (Zhang et al., 2023). In this study, a total of thirteen differentially accumulated metabolites were compared separately, including three anthocyanins (pelargonidin 3,5-di-O-glucoside, delphinidin, delphinidin 3-(6″-malonylglucoside) 5-glucoside) and ten flavonoids (kaempferol 7-(6″-galloylglucoside), 6-hydroxyluteolin, catechin 7-xyloside, quercetin 3’-O-glucuronide, catechin 7-glucuronide, quercetin 3-galactoside, quercetin 3-(2-caffeoylglucuronoside), quercetin 3-(2-galloylglucoside), luteolin 7-malonylglucoside, and isorhamnetin 3-(6″-malonylglucoside)). Comparative metabolomics revealed significantly elevated abundance of thirteen metabolites in non-waxy phenotypes relative to waxy counterparts (Fig. 5 d-g, Fig. S4). The excellent antioxidant capacity (Fig. 2) and α-glucosidase inhibitory activity (Fig. 3) of non-waxy black rice bran might be related to the higher levels of anthocyanins and flavonoids (Leonarski et al., 2024).

### 4.5 Analysis of differential metabolites in black rice bran samples

**Fig. 6:**
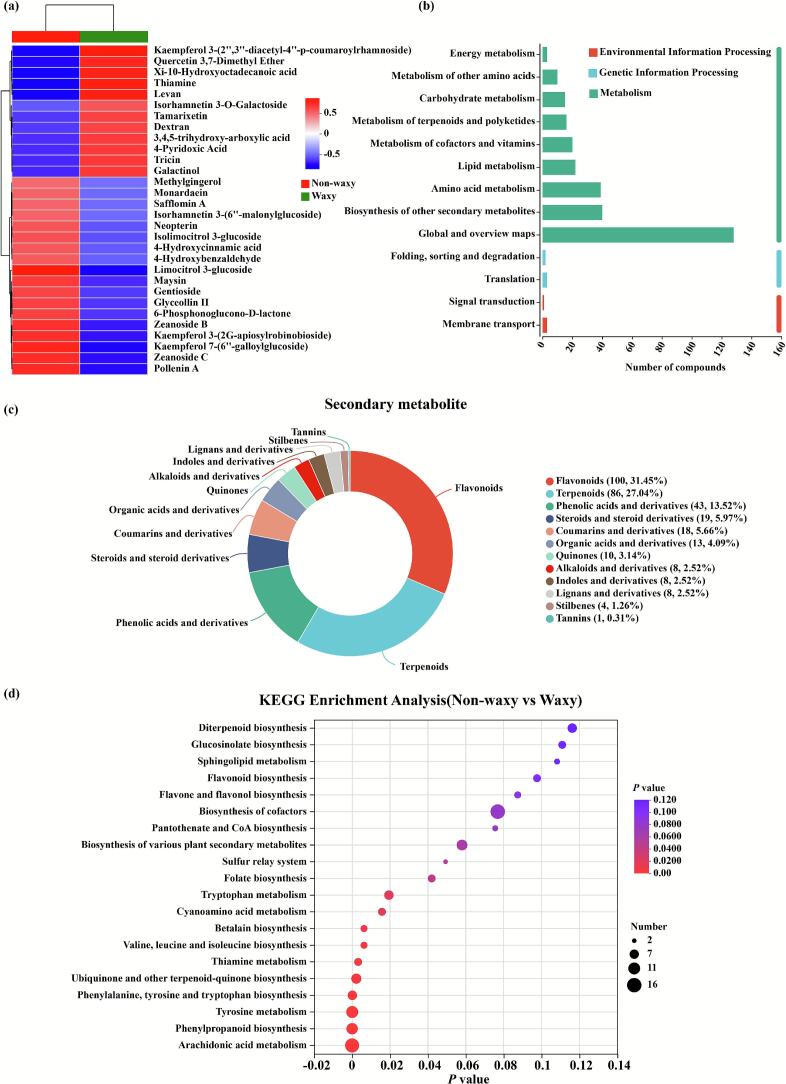
The cluster heatmap (a), KEGG pathway classification (b), classification based on plant secondary metabolites (c), and KEGG pathway enrichment analysis (d) of differential metabolites in non-waxy and waxy groups.

The metabolites with similar expression patterns usually have functional correlations (Chuang et al., 2018; Heo et al., 2022). The cluster analysis was conducted on differential metabolites (top 30) in non-waxy and waxy groups (Fig. 6 a). The results indicated that 12 and 18 metabolites were enriched in non-waxy and waxy groups, respectively. Metabolic pathway mapping via KEGG database (Kanehisa & Goto, 2000) identified differential accumulation signatures significantly enriched (*p* < 0.05) in three functional modules: metabolism, genetic information processing, and environmental signal transduction (Fig. 6 b). Importantly, the changes in metabolism pathways were the greatest, which might be a significant reason for the difference in antioxidant activity between non-waxy and waxy groups (Fig. 2, Fig. 3).

On the level of plant secondary metabolites, 318 differential metabolites were confirmed in this study, mainly involving flavonoids (31.45 %), terpenoids (27.04 %), and phenolic acids and derivatives (13.52 %) (Fig. 6 c). KEGG enrichment analysis revealed 73 significantly perturbed pathways for intergroup discriminant metabolites. Pathway impact values were visualized through enrichment bubble charts, highlighting top-ranked metabolic routes (Fig. 6 d, and Fig. S5–7). Significant enrichment (*p* < 0.05) in the non-waxy group, relative to the waxy group, was detected across diverse metabolic pathways. This included pathways central to amino acid metabolism (tryptophan, tyrosine, phenylalanine - encompassing their biosynthesis and specific metabolism), branched-chain amino acid biosynthesis (valine, leucine, isoleucine), specialized compound biosynthesis (phenylpropanoid, betalain, ubiquinone/terpenoid-quinone), essential cofactor biosynthesis/metabolism (thiamine, folate), arachidonic acid metabolism, and cyanoamino acid metabolism. Especially, three key differential metabolites (pelargonidin-3,5-diglucoside, kaempferol-7-(6″-galloylglucoside), and paeoniflorin) were quantified using targeted UPLC-MS/MS. The results showed that the significant upregulation of these metabolites in non-waxy group, fully consistent with our initial non-targeted metabolomics findings (Fig. S8). In addition, the expression levels of key genes in the phenylpropanoid and flavonoid biosynthetic pathways (e.g., *OsPAL*, *OsCHS*, *OsDFR*, and *OsANS*) were analyzed to investigate the regulatory mechanisms underlying metabolite accumulation. Results confirmed sustained high expression of these genes in non-waxy group (Fig. S9), providing transcriptional evidence for the observed flavonoid and phenolic acid accumulation.

### 4.6 Correlation analysis between biological activity and differential metabolites in black rice bran samples

**Fig. 7:**
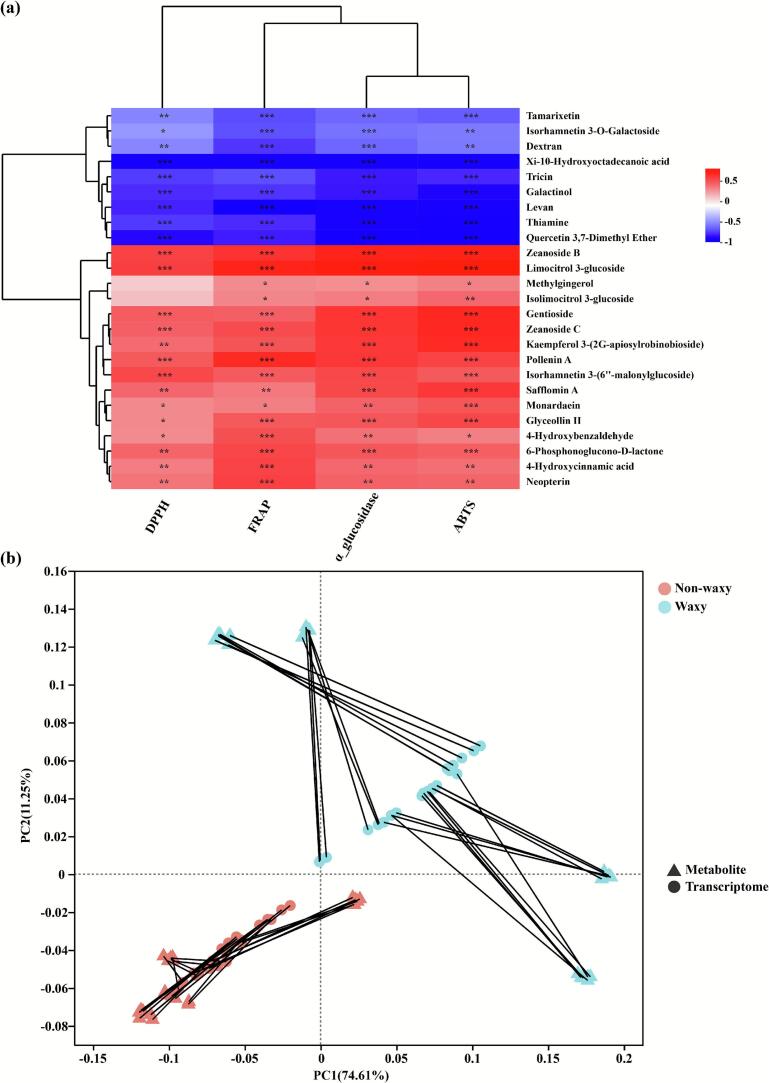
The correlation heat map (a), and procrustes analysis (b) between biological activity and differential metabolites in black rice bran samples.

To explore the relationships between biological activity and differential metabolites in black rice bran samples, the association analysis was employed in this study (Fig. 7). The correlation analysis of antioxidant capacity, α-glucosidase inhibitory activity, and the dominant metabolites (top 25 differentially expressed metabolites) were illustrated (Fig. 7 a). The correlation between ABTS radical-scavenging activity, FRAP assay, α-glucosidase inhibitory activity and differential metabolites showed astonishing similarities, with significant positive correlations with 16 compounds and significant negative correlations with 9 compounds. Additionally, the abundances of kaempferol 3-(2G-apiosylrobinobioside), isorhamnetin 3-(6″-malonylglucoside), and 4-hydroxycinnamic acid were significantly positively correlated with DPPH radical-scavenging activity, ABTS radical-scavenging activity, FRAP assay, and α-glucosidase inhibitory activity. For example, the addition of 4-hydroxycinnamic acid significantly increased the total phenolic and total anthocyanin content in wine made from Cabernet Sauvignon grapes (Wu et al., 2025), while the increase in total phenolics partially enhanced antioxidant activity. Total phenolic and total flavonoid content significantly increased during corn silk fermentation, with phenolic compounds primarily consisting of flavonoids and hydroxycinnamic acids. Simultaneously, antioxidant activity measured by ABTS, DPPH, and FRAP assays also markedly improved (Frumuzachi et al., 2025). Polyphenols in black mulberry juice enhance cellular vitality. Metabolomic analysis indicates that biotransformation of specific phenolic acids (e.g., hydroxycinnamic acid and hydroxybenzoic acid) and degradation of rutin and anthocyanins contribute to its enhanced biological activity (Lv et al., 2025). Moreover, for kaempferol 3-(2-O-apiosyl-robinobioside), high-pressure homogenization enhances quinoa's phenolic compounds, particularly O-glycosides of kaempferol (de Carvalho Oliveira et al., 2024; de Carvalho Oliveira et al., 2025). Its rich flavonoid profile confers benefits including antioxidant, anti-inflammatory, anticancer, and antibacterial properties. Therefore, these findings may help explain the variations in antioxidant activity observed under different waxy characteristics, particularly regarding phenolic compounds. However, future research should further investigate the biotransformation of polyphenolic compounds to enhance the antioxidant activity of black rice, while emphasizing this metabolic conversion mechanism. Procrustes analysis is commonly used to evaluate whether the trends in activity and metabolomics expression levels of samples between different groups are consistent (Nguyen et al., 2021; Song et al., 2025). In our study, the correlation characteristics and metabolite expression levels showed significant consistency in trends between non-waxy and waxy groups (Fig. 7 b). These findings imply that waxy characteristics may influence the differing antioxidant activities and metabolite profiles observed in the eight black rice varieties.

## 5 Conclusions

In this paper, eight typical local black rice varieties from eight provinces of China were divided into non-waxy (BR1-BR4) and waxy (BR5-BR8) groups according to the amylose content with non-waxy varieties transparent-grained with low chalkiness and waxy varieties white opaque-grained, respectively. In-depth analyses showed that non-waxy black rice bran had significant superior DPPH, ABTS radical-scavenging activities and FRAP-reducing power, increased total polyphenols (+30.75 %), and stronger α-glucosidase inhibitory activity. Extensive targeted metabolomics analysis conducted by UPLC-MS/MS revealed that there were 349 upregulated and 281 downregulated metabolites between non-waxy and waxy groups. Notably, anthocyanins (such as pelargonidin 3,5-di-O-glucoside, delphinidin) and flavonoids (e.g., kaempferol 7-(6″-galloylglucoside)) were significantly enriched in non-waxy bran, mainly due to the high expression of key genes in the phenylpropanoid and flavonoid biosynthetic pathways (*OsPAL*, *OsCHS*, *OsDFR*, *OsANS*). Correlation and Procrustes analyses revealed that metabolites including kaempferol 3-(2G-apiosylrobinobioside, isorhamnetin 3-(6″-malonylglucoside, and 4-hydroxycinnamic acid were positively linked to antioxidant and α-glucosidase inhibitory activities, which suggested that better functional activities of nonwaxy bran was due to the enhanced phenolic metabolites content. It shows that wax characteristics substantially influence the black rice bran metabolic profile and biological action, and the non-waxy black rice bran possesses the potentiality to be a source of natural antioxidants and hypoglycemic functional factors. Future studies will need to be conducted to explain the biotransformation of polyphenolic compounds and confirm the functional responses caused by non-waxy black rice bran in vivo, so as to take non-waxy black rice bran into application for functional foods.

## CRediT authorship contribution statement

**Shiquan Bian:** Writing – original draft, Methodology, Data curation. **Shaojie Song:** Writing – original draft, Resources. **Mingding Qu:** Formal analysis, Data curation. **Xiaojing Dang:** Formal analysis, Data curation. **Deyong Mei:** Formal analysis, Data curation. **Cuixiang Lin:** Visualization, Investigation. **Wanlin Wang:** Visualization, Investigation. **Jintao Shang:** Visualization, Supervision. **Dahu Ni:** Writing – review & editing, Supervision, Project administration, Funding acquisition. **Dewen Zhang:** Writing – review & editing, Supervision, Project administration, Funding acquisition.

## Declaration of competing interest

The authors declare that they have no known competing financial interests or personal relationships that could have appeared to influence the work reported in this paper.

## Data Availability

No data was used for the research described in the article.

## Appendix A Supplementary data

Supplementary material Image 1
