# Supplementary material for: Comparative analysis of the metabolite compositions and antioxidant capacity of waxy and non-waxy black rice (Oryza sativa L.) bran via widely targeted metabolomics
Source: Food Chem X. 2025 Dec 8;32:103387. doi: 10.1016/j.fochx.2025.103387 (PMC12756000; doi:10.1016/j.fochx.2025.103387)
Supplement: Supplementary file 1 — Supplementary material [file mmc1.docx]

**Comparative analysis of the metabolite compositions and antioxidant capacity of waxy and non-waxy black rice (*Oryza sativa* L.)** **bran via widely targeted metabolomics**

**The lists of supplementary materials:**

**Table S1**. The information of black rice variety samples

**Table S2.** Elution gradient of the mobile phase in C18 column

**Table S3.** Mass spectrometry parameters used in this study

**Table S4**. Primers for qRT-PCR analysis

**Fig. S1.** The amylose contents in black rice variety samples

**Fig. S2.** The total ion chromatography (TIC) in black rice variety samples based on UPLC-MS/MS

**Fig. S3.** The total ion chromatography (TIC) in non-waxy and waxy groups based on UPLC-MS/MS

**Fig. S4.** The comparison of metabolic profiles in non-waxy and waxy groups

**Fig. S5.** The KEGG topology analysis of differential metabolites in non-waxy and waxy groups

**Fig. S6.** The KEGG multidimensional enrichment circle diagram of differential metabolites in non-waxy and waxy groups

**Fig. S7.** The KEGG enrichment analysis network diagram of differential metabolites in non-waxy and waxy groups

**Fig. S8.** The comparison of three key differential metabolites in non-waxy and waxy groups using targeted UPLC-MS/MS

**Fig. S9.** The comparison of the expression levels of key genes in the phenylpropanoid and flavonoid biosynthetic pathways

**Table S1. The information of black rice variety samples**

| Variety name | Province | TGW (thousand-grain weight, g) |
| --- | --- | --- |
| BR1 (Yimengheimi) | Shandong Province | 20.83±0.25 |
| BR2 (Jiagouheimi) | Anhui Province | 26.32±0.25 |
| BR3 (Heizhenzhu) | Heilongjiang Province | 22.05±0.13 |
| BR4 (Mojiangzimi) | Yunnan Province | 25.01±0.42 |
| BR5 (Yangxianheigu) | Shanxi Province | 23.36±0.57 |
| BR6 (Bamaheimi) | Guangxi Province | 22.26±0.12 |
| BR7 (Zixiangnuo) | Xinjiang Province | 38.36±1.30 |
| BR8 (Ziquezimi) | Hunan Province | 24.02±0.35 |

**Table S2. Elution gradient of the mobile phase in C18 column**

| Time (min) | Flow rate (mL/min) | Mobile phase A (%) | Mobile phase B (%) |
| --- | --- | --- | --- |
| 0.0 | 0.4 | 98 | 2 |
| 0.5 | 0.4 | 98 | 2 |
| 7.5 | 0.4 | 65 | 35 |
| 13.0 | 0.4 | 5 | 95 |
| 14.4 | 0.4 | 5 | 95 |
| 14.5 | 0.4 | 98 | 2 |
| 16.0 | 0.4 | 98 | 2 |

**Table S3. Mass spectrometry parameters used in this study**

| Description | Parameter |
| --- | --- |
| Scan type (m/z) | 70-1050 |
| Sheath gas flow rate (arb) | 50 |
| Aux gas flow rate (arb) | 13 |
| Heater temp (℃) | 450 |
| Capillary temp (℃) | 320 |
| Spray voltage (+) (V) | 3500 |
| Spray voltage (-) (V) | -3000 |
| S-Lens RF Level | 40 |
| Normalized collision energy (%) | 20,40,60 |
| Resolution (Full MS) | 70000 |
| Resolution (MS2) | 17500 |

**Table S4. Primers for qRT-PCR analysis**

| GenBank Accession | Genes | Primer Sequence |
| --- | --- | --- |
| X16099 | OsPAL-F | GAGAAGAACCTCCTCACCGC |
|  | OsPAL-R | ATCTTGGAGAACACGGAGGC |
| X89859 | OsCHS-F | GGGGCTCATCTCGAAGAACA |
|  | OsCHS-R | GACATGTTGCCGTACTCGGA |
| Y07956 | OsDFR-F | AACGAGGTGATCAAGCCCAC |
|  | OsDFR-R | GAAGTCGATGTCGCTCCAGT |
| Y07955 | OsANS-F | AGCTGCTCGCCATCCTCTCC |
|  | OsANS-R | GCTGACGTCGGTGTGTGCC |
| X16280 | OsActin-F | CATTGGTGCTGAGCGTTTCC |
|  | OsActin-R | TCAGCAATGCCAGGGAACAT |

**
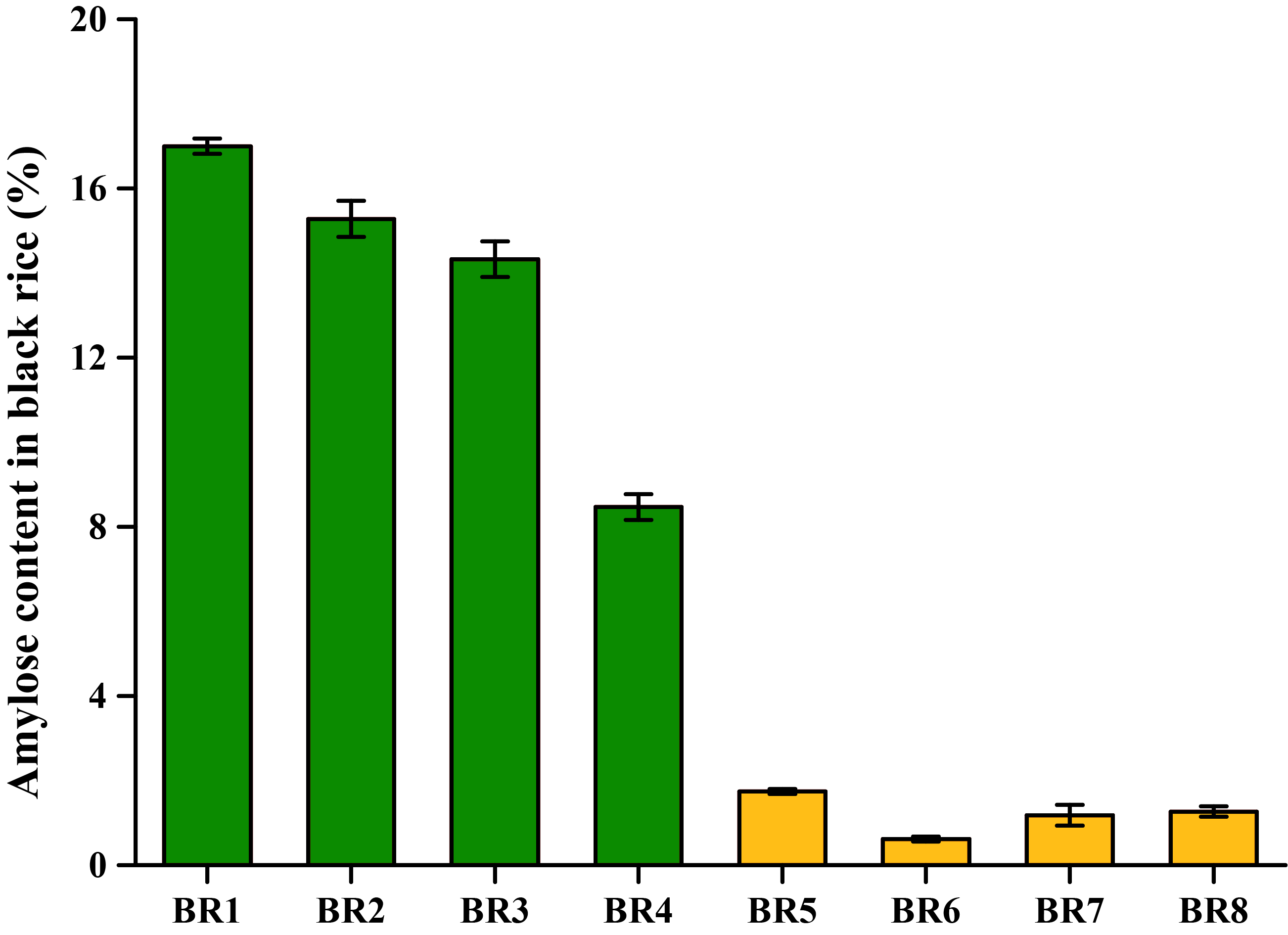
**

**Fig. S1. The amylose contents in black rice variety samples.**

**
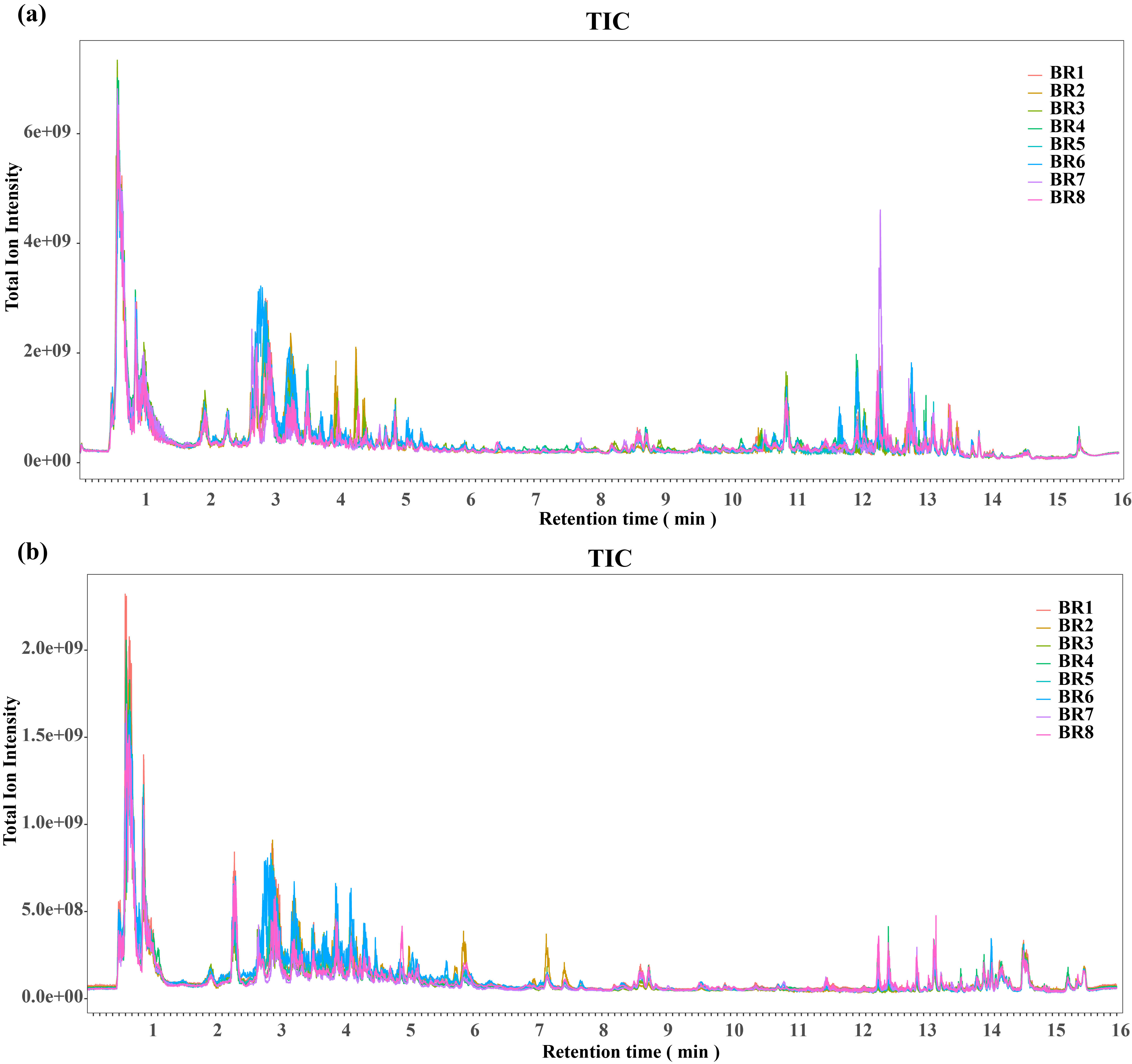
**

**Fig. S2. The total ion chromatography (TIC) in black rice variety samples based on UPLC-MS/MS. (a) positive ion mode, (b) negative ion mode.**


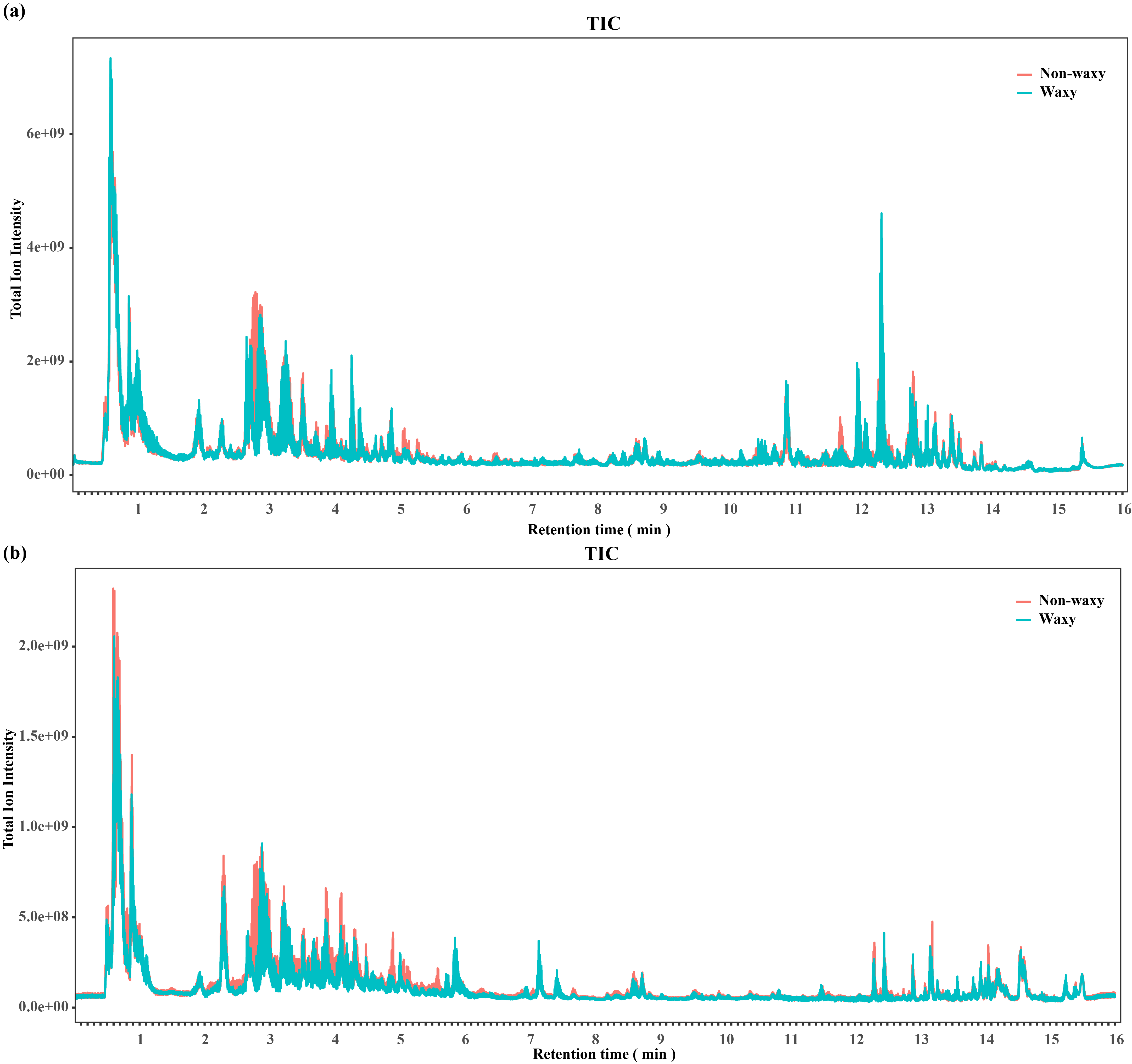


**Fig. S3. The total ion chromatography (TIC) in non-waxy and waxy groups based on UPLC-MS/MS. (a) positive ion mode, (b) negative ion mode.**

**
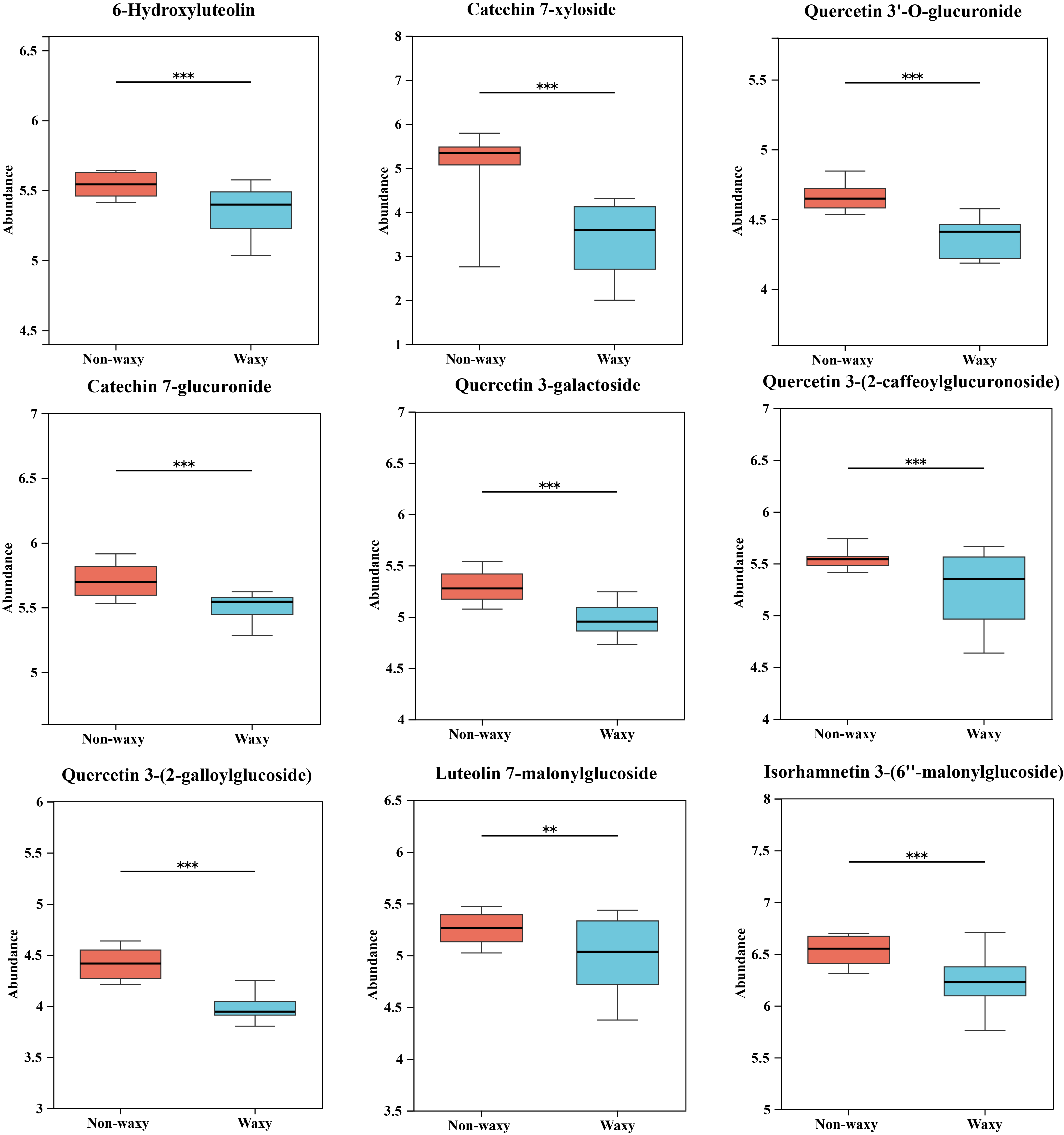
**

**Fig. S4. The comparison of metabolic profiles in non-waxy and waxy groups.**

**
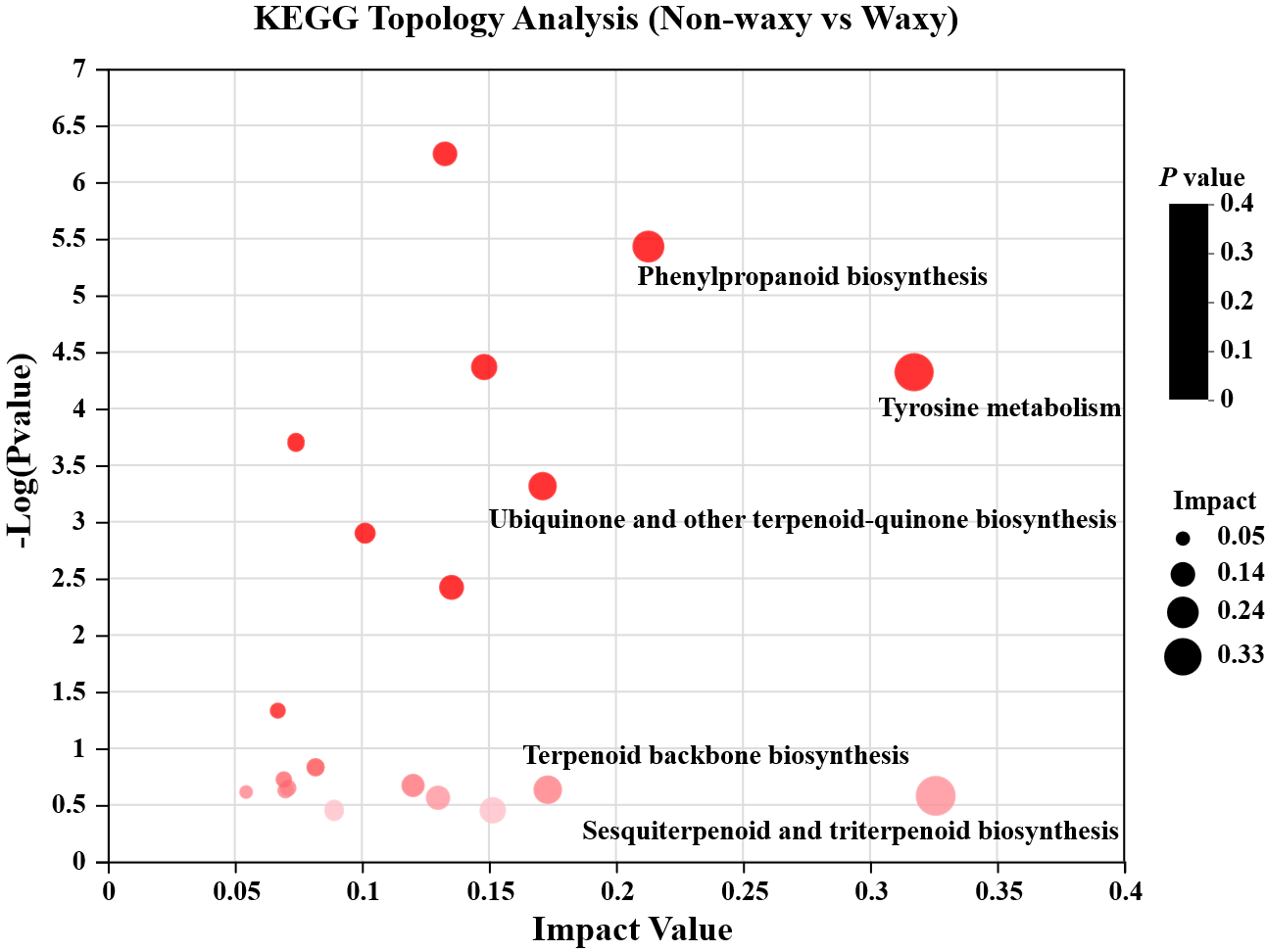
**

**Fig. S5. The KEGG topology analysis of differential metabolites in non-waxy and waxy groups.**

**
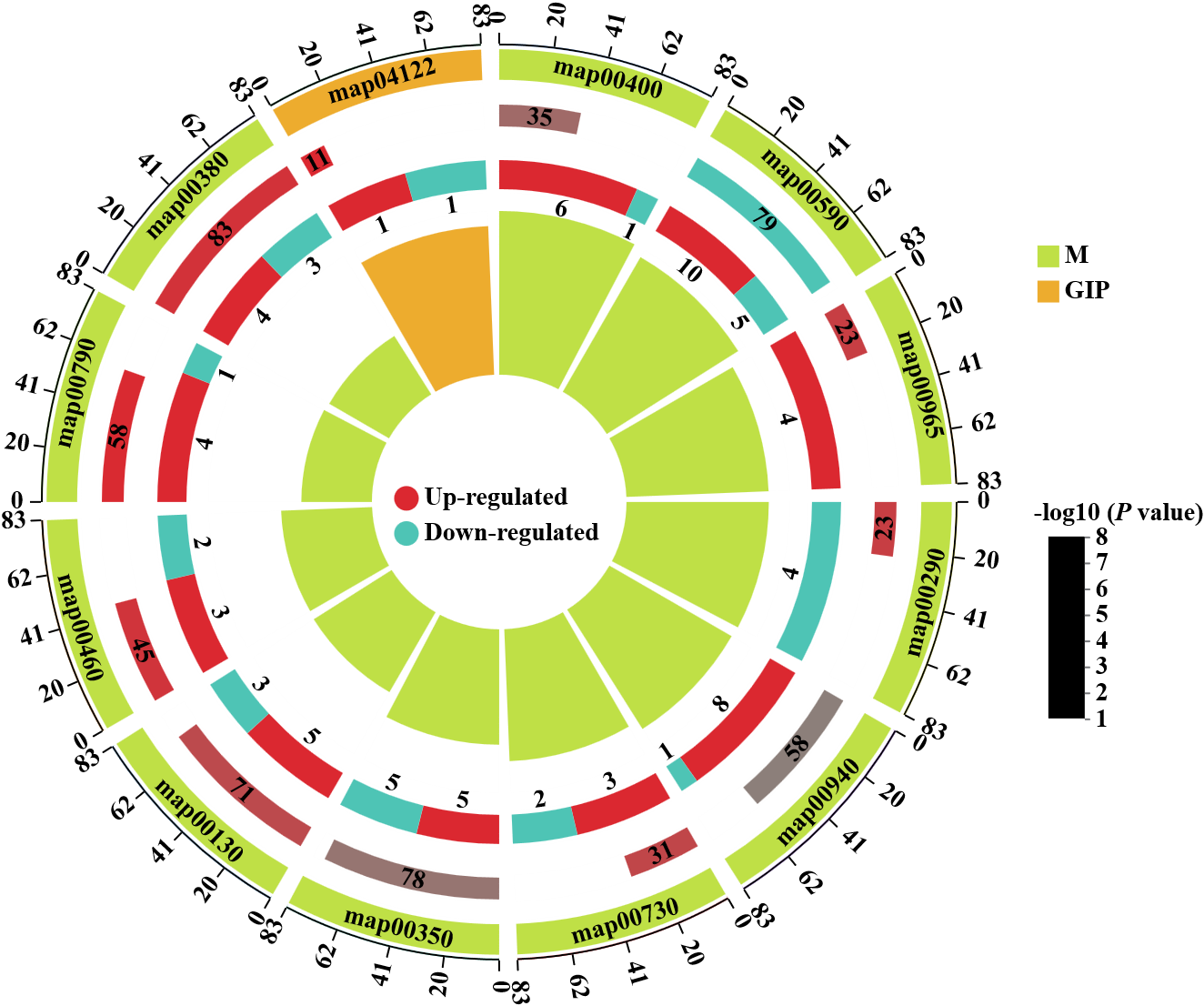
**

**Fig. S6. The KEGG multidimensional enrichment circle diagram of differential metabolites in non-waxy and waxy groups.**

**
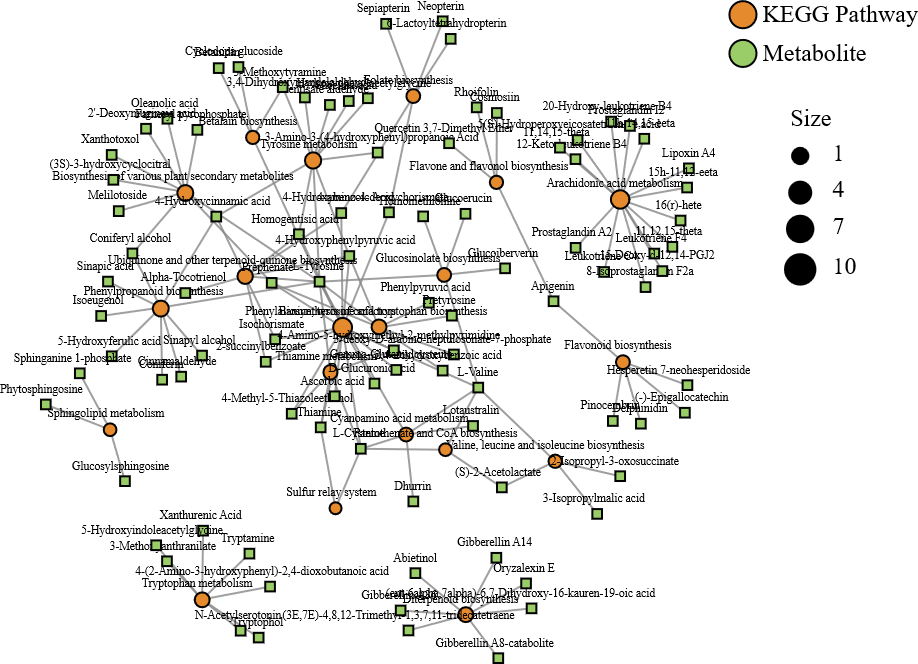
**

**Fig. S7. The KEGG enrichment analysis network diagram of differential metabolites in non-waxy and waxy groups.**

**
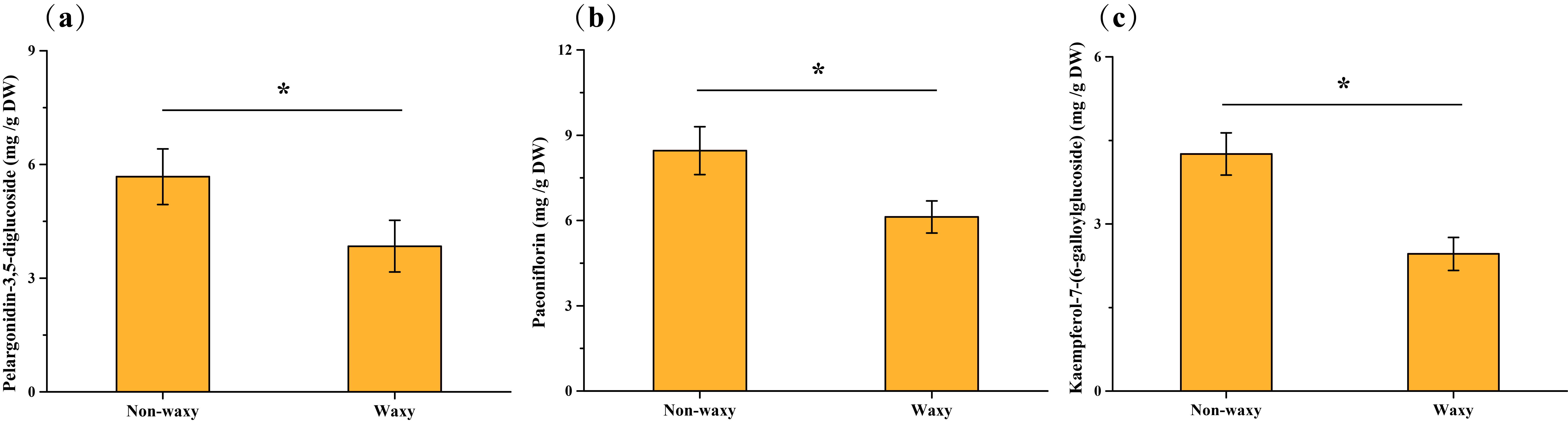
**

**Fig. S8. The comparison of three key differential metabolites in non-waxy and waxy groups using targeted UPLC-MS/MS.** **The * indicates a significant difference between waxy and non-waxy black rice bran (*p* < 0.05).**

**
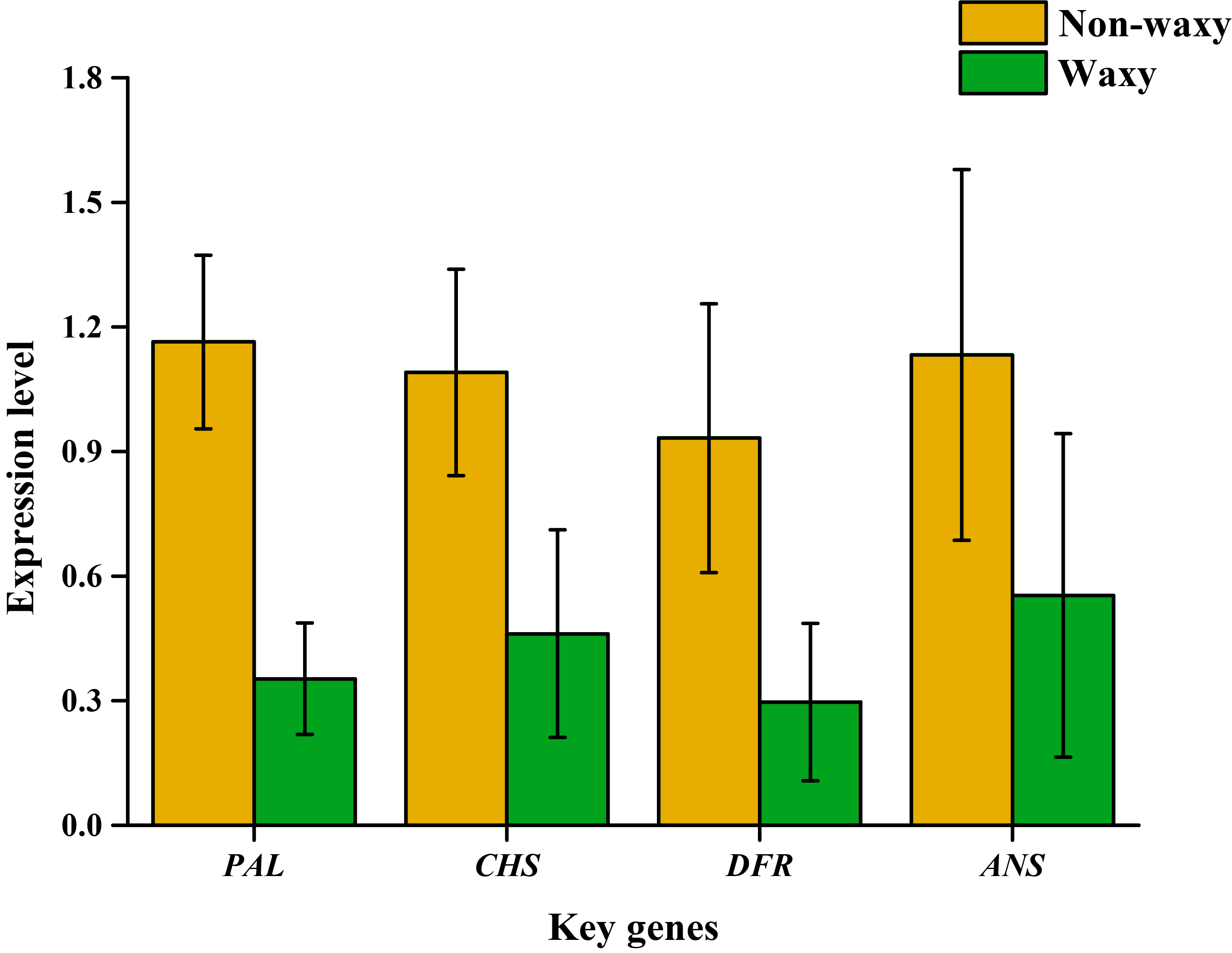
**

**Fig. S9. The comparison of the expression levels of key genes in the phenylpropanoid and flavonoid biosynthetic pathways.**
